# Regulation of the cerebral circulation: bedside assessment and clinical implications

**DOI:** 10.1186/s13054-016-1293-6

**Published:** 2016-05-05

**Authors:** Joseph Donnelly, Karol P. Budohoski, Peter Smielewski, Marek Czosnyka

**Affiliations:** Brain Physics Laboratory, Division of Neurosurgery, Department of Clinical Neurosciences, Cambridge Biomedical Campus, University of Cambridge, Hills Road, Cambridge, CB2 0QQ UK; Institute of Electronic Systems, Warsaw University of Technology, ul. Nowowiejska 15/19, 00-665 Warsaw, Poland

## Abstract

Regulation of the cerebral circulation relies on the complex interplay between cardiovascular, respiratory, and neural physiology. In health, these physiologic systems act to maintain an adequate cerebral blood flow (CBF) through modulation of hydrodynamic parameters; the resistance of cerebral vessels, and the arterial, intracranial, and venous pressures. In critical illness, however, one or more of these parameters can be compromised, raising the possibility of disturbed CBF regulation and its pathophysiologic sequelae. Rigorous assessment of the cerebral circulation requires not only measuring CBF and its hydrodynamic determinants but also assessing the stability of CBF in response to changes in arterial pressure (cerebral autoregulation), the reactivity of CBF to a vasodilator (carbon dioxide reactivity, for example), and the dynamic regulation of arterial pressure (baroreceptor sensitivity). Ideally, cerebral circulation monitors in critical care should be continuous, physically robust, allow for both regional and global CBF assessment, and be conducive to application at the bedside. Regulation of the cerebral circulation is impaired not only in primary neurologic conditions that affect the vasculature such as subarachnoid haemorrhage and stroke, but also in conditions that affect the regulation of intracranial pressure (such as traumatic brain injury and hydrocephalus) or arterial blood pressure (sepsis or cardiac dysfunction). Importantly, this impairment is often associated with poor patient outcome. At present, assessment of the cerebral circulation is primarily used as a research tool to elucidate pathophysiology or prognosis. However, when combined with other physiologic signals and online analytical techniques, cerebral circulation monitoring has the appealing potential to not only prognosticate patients, but also direct critical care management.

## Background

To function, the brain requires adequate delivery of nutrients and oxygen. A circulatory system is therefore required to maintain an optimal cerebral blood flow (CBF) for the brain’s diverse needs. Whilst oxygen and nutrient delivery is in part dependent on the pump supplying it—the heart—the circulatory system has also evolved mechanisms to ensure the precise control of CBF. The cerebral vessels have the remarkable ability to rapidly adapt and react to the brain’s chemical environment, to neuronal signals, and to the pressure within the cerebral vessels.

This review highlights clinically relevant aspects of cerebrovascular physiology and cerebral circulation monitoring techniques before outlining the state of current knowledge of the cerebral circulation in selected critical illnesses and highlighting promising areas for future research.

## Review

### Regulation of cerebral blood flow

**Fig. 1:**
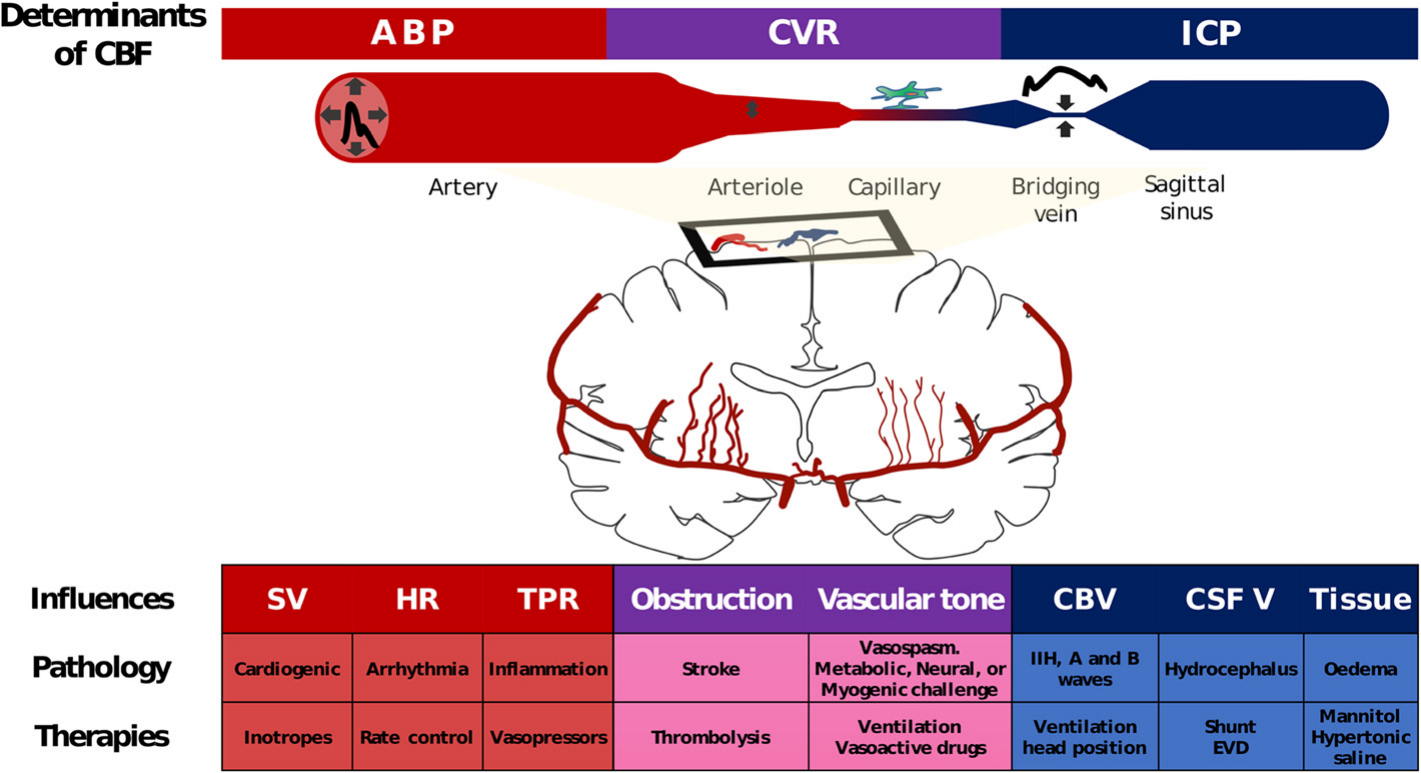
Regulation of the cerebral circulation. CBF at the level of the microvasculature is directly proportional to CPP (difference between ABP and ICP) and inversely proportional to CVR. ICP exerts its effect on CBF through changes in CPP; compression of the venous vasculature where the bridging veins enter the sagittal sinus ensures that the bridging vein and post-capillary intravascular pressure is always above ICP. CBF is modulated by the cardiovascular system in terms of the regulation of SV, HR, and TPR (*red*). Control of TPR with vasopressors forms an integral part of many CBF protective strategies (even when TPR is not the primary cause of CBF disturbance). CVR is regulated at the level of the arterioles (*purple*) by variations in vascular tone in response to metabolic, neural, or myogenic inputs. In ischaemic stroke or vasospasm, CVR is dramatically increased, usually at the level of large intracranial arteries. ICP (*blue*) modulates CBF through its coupling with cerebral venous pressure. ICP increases can be caused by increases in cerebral blood volume (arterial or venous), increased CSF volume or increase in parenchyma (oedema), or abnormal material volume (mass lesion). All therapies that modulate CBF do so via one (or more) of these pathways. There is typically significant interdependence between the therapies, determinants, and influences of CBF. For example, a drop in ABP would be expected to result in a drop in CBF but this is short lived due to the baroreflex (HR increase in response to drop in ABP) and cerebral autoregulation (decrease in vascular tone in response to drop in ABP). *ABP* arterial blood pressure, *CBF* cerebral blood flow, *CBV* cerebral blood volume, *CSF V* cerebrospinal fluid volume, *CVR* cerebrovascular resistance, *EVD* external ventricular drainage, *HR* heart rate, *ICP* intracranial pressure, *IIH* idiopathic intracranial hypertension, *SV* stroke volume, *TPR* total peripheral resistance

A haemodynamic model for the cerebral circulation has been described that allows for interrogation of the regulation of CBF [1, 2]. In such a model, CBF is dependent on the pressure supplied in the cerebral arteries (arterial blood pressure (ABP)), the back pressure in the cerebral venous system (usually close to intracranial pressure (ICP)), and the resistance related to the diameter of the small cerebral vessels (cerebrovascular resistance (CVR); Fig. 1). This relationship can be simplified as:

$$ CBF=\frac{ABP-ICP}{CVR} $$

Thus, cardiovascular, ICP, and cerebrovascular components are all important regulators of the cerebral circulation. Applying this model can provide crucial insights into the physiologic factors that regulate cerebral perfusion in health and elucidate why CBF regulation is often impaired in pathologic states.

#### The cardiovascular component

As early as 1890, Sherrington and Roy underlined the importance of the ABP in the regulation of CBF: ‘One of the most evident of the facts observed by us is that the blood-supply of the brain varies directly with the blood pressure in the systemic arteries’ [3]. The pressure that supplies the cerebral vessels is dependent on factors mostly outside the brain itself: the heart provides the cardiac output while the peripheral vessels provide the resistance, both of which contribute to the ABP supplying the brain. In this sense, the balance between the brain CVR and the total peripheral resistance determines the proportion of the cardiac output that reaches the brain. Thus, any pathological or physiological event that affects the heart or the vasculature as a whole has the potential to alter the cerebral circulation. Cardiogenic shock and arrhythmia may therefore impair CBF [4], as do conditions that affect the systemic vasculature such as sepsis [5].

Just as pathologies affecting ABP can affect CBF, therapies to augment CBF often do so by modulating ABP. Vasopressors act to buffer ABP by constricting peripheral vessels, while inotropes act to modulate cardiac output (Fig. 1). An important consideration of such an approach is that the relationship between changes in ABP and CBF is typically non-linear due to active changes in vascular tone occurring at the level of the cerebral arterioles—a process known as cerebral autoregulation (see later). Furthermore, modulating ABP as a therapeutic measure will not only increase blood flow to the brain, but will also increase blood flow to any vascular beds with a low vascular resistance.

#### The intracranial pressure component

At the distal end of the microvasculature is the cerebral venous pressure, which provides a back pressure that may impede CBF. The venous pressure in turn will be related to both the venous pressure in the larger cerebral veins and the ICP. If the ICP is above the pressure in the lateral lacunae that feed into the large venous sinuses (which are exposed to the cerebrospinal fluid (CSF) space; Fig. 1), then these vessels will be compressed leading to a post-capillary venous pressure just above the ICP [6, 7].

Any increase in ICP has the potential to decrease the longitudinal pressure gradient across the vascular bed—the cerebral perfusion pressure (CPP = ABP – ICP)—and, provided there are no compensatory changes in CVR, to decrease CBF. Thus, CBF is impaired by conditions that impede cerebral venous outflow (such as idiopathic intracranial hypertension or neck position) and by conditions that increase ICP (such as the oedema associated with traumatic brain injury (TBI) or subarachnoid haemorrhage (SAH)).

Because the skull is rigid, any increase in volume of a brain compartment can cause an increase in ICP. Increases in volume of the intravascular compartment, the CSF compartment, or the brain parenchymal compartment can all increase ICP and therefore decrease CBF. These compartmental volume changes could be caused by vascular dilation, hydrocephalus, or cerebral oedema. Therapies that alter CBF via ICP changes include mild hyperventilation to decrease vascular volume, CSF diversion through external ventricular drainage to decrease CSF volume, osmotherapy to reduce the brain tissue volume, or decompressive craniectomy to increase the space available for the brain parenchyma (Fig. 1).

#### The cerebrovascular component

At the level of the brain vessels themselves, CBF can be controlled by active changes in the diameter of the ‘regulating’ vessels, thus influencing the CVR.

The major site of active regulation of the cerebral circulation is thought to be at the level of the arterioles with their thick smooth muscle layer and ability for profound dilation and constriction [1, 2]. However, larger conduit arteries, capillaries, and venous structures may also be important in certain situations [8–11]. For example, during neuronal activation, relaxation of pericytes surrounding capillaries has been considered to account for a large proportion of the flow increase [9]. Cerebral venules and veins are characterised by a low density of smooth muscle cells and therefore have the ability to increase volume with any increase in pressure; that is, they exhibit a high compliance [11]. While probably not important in the active regulation of CBF, the compliant nature of venous structures may play a passive role in the regulation of CBF; for example, arteriolar dilation leads to an increase in the volume of post-capillary venules that increases cerebral blood volume [12] and by extension could increase ICP, decrease CPP, and therefore limit the increase in CBF.

In health, such changes in CVR or CBF are most obvious during brain activation; an increase in neuronal activity elicits a prompt and significant increase in CBF [13] mediated through vessel dilation. Alternatively, during an ischaemic stroke, a portion of the cerebral vasculature is mechanically occluded by a thrombus causing a localised increase in CVR and a decrease in CBF. During the vasospasm associated with SAH, large cerebral arteries constrict, again resulting in an increased local CVR and decreased CBF [14].

Changes in vascular tone of the cerebral vessels are caused by putative constricting and dilating substances. Such vasoactive substances may be supplied to the vessels via the bloodstream (e.g. arterial pressure of carbon dioxide (PaCO_2_)), produced locally (see ‘Neurovascular coupling’), or reach the smooth muscle fibres through direct autonomic innervation. Not surprisingly, this heterogeneity in the possible sites of vasoactive substance production can lead to difficulty in disentangling physiological mechanisms. For example, modulation of ventilation is commonly used to assess the function of the cerebral vasculature (see ‘Carbon dioxide reactivity’); however, such a stimulus can in principle alter cerebrovascular tone through three separate mechanisms: changes in PaCO_2_ reaching the brain [15], changes in autonomic activity [16], or direct changes in neuronal activity [17].

Synaptic transmission with its resulting glutamate release is the important stimulus for neurovascular coupling through the production of vasoactive metabolites such as arachidonic acid derivatives (20-hydroxy-eicosatetraenoic acid, prostaglandins, epoxyeicosatrienoic acids), lactate, adenosine and nitric oxide [8]. The site of production of these metabolites includes the neuron, the astrocyte, and the smooth muscle cells themselves. Both neurons and astrocytes are ideally placed to mediate neurovascular coupling as they lie in close proximity to both the neuronal synapse where the signal is initiated and the smooth muscle cells of the regulating microvasculature; however, the relative importance of neurons versus astrocytes for neurovascular coupling is uncertain [8]. Regardless of the site of production, the site of action is the smooth muscle fibres surrounding the arterioles, or capillaries where the vasoactive substances produce changes in intracellular calcium concentration, which in turn alters the degree of smooth muscle contraction, and vessel constriction. For further review on neurovascular coupling, see [8, 18–22].

The autonomic nervous system may also influence the vascular tone of cerebral vessels. Despite animal studies demonstrating a rich innervation of both the dilating parasympathetic and constricting sympathetic fibres, the autonomic control of CBF in humans remains controversial [23, 24] with the divergence in opinions probably owing to between-species variation in autonomic innervation, variations in brain metabolism between experiments, and heterogeneous autonomic nerve distribution in the different studies [25]. Nevertheless, stimulation of the trigeminal ganglion in humans decreases the estimated CBF [26] while blockade of the stellate ganglion increases the estimated CBF [27], highlighting a role for the sympathetic nervous system in the regulation of the cerebral circulation in humans.

In addition to the cerebrovascular, mean arterial pressure, and ICP components, cardiac output has recently been suggested to be an independent regulator of CBF [28]. Evidence for such a view comes from studies demonstrating a change in CBF after interventions that change cardiac output but have no effect on mean arterial pressure [28, 29]. An additional measure of CBF regulation could thus be assessing CBF as a fraction of the cardiac output. Although continuous and accurate measures of cardiac output are less practical than ABP, such an approach may provide additional insight into regional blood flow regulation in health and disease.

According to the conventional model (Fig. 1), for an increase in cardiac output to produce an increase in CBF without a change in ABP, both total peripheral resistance and CVR must decrease. As such, the autonomic nervous system has been speculated as the mechanism by which changes in cardiac output may alter CBF without changes in ABP [28]; however, a metrological issue should also be considered. The ABP measured in the examined studies (and the majority of vascular regulation investigations) is not the ABP in the large cerebral arteries, but the pressure in a small peripheral vessel or that estimated non-invasively at the finger or arm. Thus, in situations where an increase in cardiac output causes an increased CBF and seemingly unchanged ABP (estimated at the arm), it is possible that cerebral arterial pressure actually increases. This issue needs to be verified, probably in an animal model.

Finally, the simple schema provided in Fig. 1 must be interpreted with the knowledge of the interdependence of variables. The cerebral circulation appears to have several cerebroprotective mechanisms; for example, if ABP decreases, aortic and carotid baroreceptors will alter autonomic outflow to increase HR and therefore buffer ABP and CBF [30]. Similarly, as proposed by Lassen and elaborated upon by others, in response to a decrease in ABP, vessels will dilate in attempt to buffer CBF [31, 32]. These important cerebroprotective processes are known as baroreceptor sensitivity and cerebral autoregulation.

### How to assess the regulation of cerebral blood flow

Given the importance of CBF regulation in many pathological states, the availability of accurate and practical assessment methodologies is crucial. Often the choice of an appropriate measurement technique depends upon the clinical need; a balance between availability, accuracy, and practicality must be reached.

Non-invasive monitoring techniques include transcranial Doppler (TCD) and near-infrared spectroscopy (NIRS) (for a recent review, see [33, 34]). Such modalities have several important advantages making them suitable for interrogating CBF regulation in the clinical setting (Table 1). First, both TCD and NIRS systems are portable and non-invasive, making assessment feasible in the emergency room, the critical care unit, or the operating theatre. Moreover, they capture high-frequency and continuous data that can be combined with other modalities (such as ABP or end-tidal carbon dioxide (CO_2_)) to give information on cerebral autoregulation and CO_2_ reactivity (see ‘Carbon dioxide reactivity’).

**Table 1:** Clinical assessment methodologies for the cerebral circulation

Method	Principle	Global or local CBF assessment	Robustness	Invasive	Bedside	Continuous	Advantage	Disadvantage
TCD [33]	Doppler principle	Global (vascular territory)	Fair	No	Yes	Yes	High-frequency signal	Signal easily lost. Flow velocity assessment only
NIRS [34]	Absorbance of oxygenated and deoxygenated haemoglobin	Local	Good	No	Yes	Yes	Easy application	Uncertain intracranial contribution to signal
P_B_TO_2_ [37]	Clark electrode	Local	Excellent	Yes	Yes	Yes	Robust	Local
LDF [36]	Doppler principle	Local	Excellent	Yes	Yes	Yes	Assessment of microcirculation	Unknown biological zero
Thermal diffusion [35]	Thermal diffusion	Local	Excellent	Yes	Yes	Yes	Absolute CBF	Frequent calibrations
Duplex neck US [106]	Doppler principle	Global	Poor	No	Potentially	No	Absolute and global CBF	Semi-continuous
CT [107]	Time-dependent attenuation of iodine IV contrast bolus (perfusion CT) or Xe gas	Global and local	Excellent	No	Potentially	No	Global and regional CBF	Bulky and intermittent
PET [108]	Radioactive tracers emit positrons dependent on perfusion	Global and local	Excellent	Minimal (venous access)	No	No	Regional CBF and metabolism	Radiation, requires a cyclotron
MRI [109]	Perfusion-dependent decrease in T2 signal with gadolinium	Global and local	Excellent	Minimal (IV access) or no for arterial spin labelling technique	No	No	Absolute, regional and global CBF	Time-consuming, expensive, difficult to assess critically ill patients

*CBF* cerebral blood flow, *CT* computerised tomography, *IV* intravenous, *LDF* laser Doppler flowmetry, *MRI* magnetic resonance imaging, *NIRS* near-infrared spectroscopy, *P*_*B*_ *TO*_*2*_ pressure of brain tissue oxygen, *PET* positron emission tomography, *TCD* transcranial Doppler, *US* ultrasound

Invasive cerebral perfusion methods include brain tissue oxygen monitoring, laser Doppler flowmetry, and thermal diffusion (for review of methodology principles, see [35–37]). Whilst obviously only suitable for critically ill patients because of their invasive nature, these methods have the advantage of being relatively robust for long-term monitoring of the cerebral circulation. Brain imaging techniques (computerised tomography (CT), positron emission tomography, and magnetic resonance imaging) have the advantage of offering a high spatial resolution of CBF data and the ability to asses absolute CBF, but are at present not suitable for bedside monitoring because of size, temporal resolution, and radiation exposure [38].

#### Extended assessment of cerebral blood flow regulation

Because of the interdependence of the factors controlling CBF, it is important to measure these factors (ABP and ICP) in addition to CBF. Further, one can assess the regulation of the system by assessing the efficiency of the cardiac maintenance of ABP through the baroreflex sensitivity and assessing the brain vascular reactivity using the CBF reactivity to a vasodilator stimulus (CO_2_ reactivity), to a perfusion pressure challenge (cerebral autoregulation), or to a burst of neuronal activity (neurovascular coupling). Such extended assessment allows for a comprehensive understanding of the vulnerability of a patient’s cerebral circulation.

#### Carbon dioxide reactivity

The cerebral vasculature is exquisitely sensitive to changes in the PaCO_2_: with a decrease in pressure of carbon dioxide (PCO_2_), cerebral resistance vessels constrict; and with an increase in PaCO_2_, cerebral vessels dilate [15]. These alterations in vascular tone are probably mediated by changes in extracellular hydrogen ion concentration resulting from diffusion of PCO_2_ from inside the vessels. Several lines of evidence indicate that cerebrovascular reactivity may be a non-invasive and practical marker of cerebrovascular health (see ‘Clinical applications of bedside assessment of CBF regulation’).

The CO_2_ reactivity of cerebral vessels can be conveniently assessed at the bedside by measuring the CBF response to a decrease in PaCO_2_ produced by hyperventilation or to an increase in PaCO_2_ from hypoventilating or adding inspired CO_2_ (hypercapnia). Typically, CO_2_ reactivity is measured as the change in CBF as a fraction of the change in PaCO_2_:

$$ Cerebrovascular\;C{O}_2=\frac{\varDelta CBF\left(\%\right)}{\varDelta PaC{O}_2\left( mm\;Hg\right)} $$

An important consideration is that changes in PaCO_2_ may also affect ABP or ICP and therefore changes in PaCO_2_ may alter CPP in addition to CVR. In the ideal monitoring scenario, therefore, one would monitor CBF (perhaps using TCD), ABP (using an invasive arterial line or non-invasive photoplethysmography device), PaCO_2_ (or end-tidal CO_2_ as a surrogate), and in some situations ICP.

**Fig. 2:**
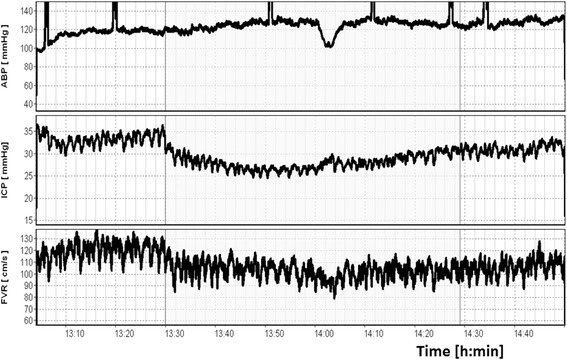
CO_2_ reactivity after TBI. CO_2_ reactivity is a measure indicating how well vascular responses in the brain are preserved. Mild hyperventilation (PaCO_2_ challenge from 35 to 31.5 mmHg) is applied temporarily (1 h) in the patient after TBI. Right CBF velocity (*FVR*) in the middle cerebral artery decreased from 120 to 100 cm/s. CO_2_ reactivity is calculated as ∆CBF velocity (%)/∆ PaCO_2_ and in this case reactivity is ~ 5 %/mmHg—very good. However, at the same time ICP decreased from 32 to 27 mmHg and blood pressure (ABP) increased from 120 to 125 mmHg. Therefore, CPP increased from 88 to 98 mmHg. The formula for cerebrovascular CO_2_ reactivity does not take into account the possible interaction between chemoregulation and autoregulation. *ABP* arterial blood pressure, *ICP* intracranial pressure

Figure 2 demonstrates a CO_2_ reactivity test in a TBI patient. In this case, the TCD-based flow velocity (Fv) was measured during moderate hyperventilation aimed to make the patient mildly hypocapnic. An important consideration easily appreciated from Fig. 1 is that during a CO_2_ reactivity test, any CO_2_ influence on ABP or ICP may confound interpretation.

#### Cerebral autoregulation

**Fig. 3:**
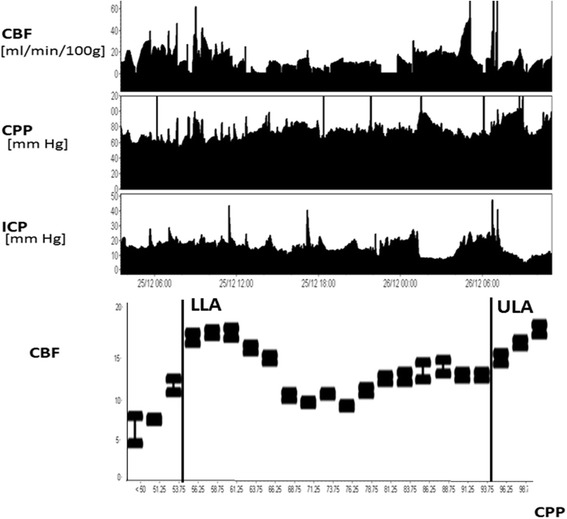
Long-term invasive CBF and CPP monitoring. Example of the ‘Lassen curve’ depicting the relationship between CPP and CBF. It is derived from a long-term plot of thermal-dilution CBF and CPP monitored in a patient after severe brain injury. The curve shows lower (*LLA*) and upper (*ULA*) limits of autoregulation, outside which CBF is pressure passive. Notably, within the autoregulation range, CBF is not ideally stable but shows an increase in CBF around the LLA, which is commonly observed in patients under mild hyperventilation (in this case PaCO_2_ was on average 32 mmHg). *CBF* cerebral blood flow, *CPP* cerebral perfusion pressure, *ICP* intracranial pressure

While cerebrovascular CO_2_ reactivity assessment attempts to gain insight into vascular function from the response of cerebral vessels to changes in PaCO_2_, cerebral autoregulation assessment attempts to gain insight into vascular function from the response of cerebral vessels to changes in ABP (or in some cases CPP). In some cases, where ABP or CPP is highly variable, the cerebral autoregulation phenomenon can be observed by plotting CBF averaged in groups of ABP or CPP (see Fig. 3). Such dramatic swings in ABP or CPP are not always observed, however, and therefore a typical assessment of cerebral autoregulation involves inducing an ABP stimulus and measuring the response of CBF. In clinical scenarios, CBF is measured before and after a vasopressor is used to augment ABP to give a point estimate of cerebral autoregulation.

An alternative approach is to monitor continuously the CBF response to natural slow variations in ABP [39]. Such an approach has some important caveats: the natural ABP variations may not be strong enough to challenge CBF, and changes in CBF could be caused by factors other than ABP. However, the monitoring poses no risk to the patients and has the distinct advantage that it can assess long-term trends in cerebral autoregulation within a patient.

The simplest methods of monitoring cerebral autoregulation assess how the slow changes of ABP occurring in time compare with the slow changes in CBF (for review, see [32]). An example of this is the mean flow index (Mx), which measures the correlation between 30 consecutive 10-s averages of TCD mean CBF velocity and CPP [40]. Methods using the frequency spectrum of the signals are also available. By assuming that the cerebral circulation acts as a high-pass filter (high-frequency fluctuations in ABP pass through to Fv unimpeded whilst lower frequencies are dampened), transfer function methods assess cerebral autoregulation using the phase (shift in degrees required to align slow waves of ABP and CBF velocity), gain (dampening factor), and coherence (degree of association between ABP and Fv) [41]. NIRS can also be used for assessment of cerebral autoregulation in the time and frequency domain and is easier to apply in many situations (less operator dependency compared with TCD). NIRS-based autoregulation indices assess the relationship between CPP (or ABP) and NIRS-based cerebral oxygenation.

**Fig. 4:**
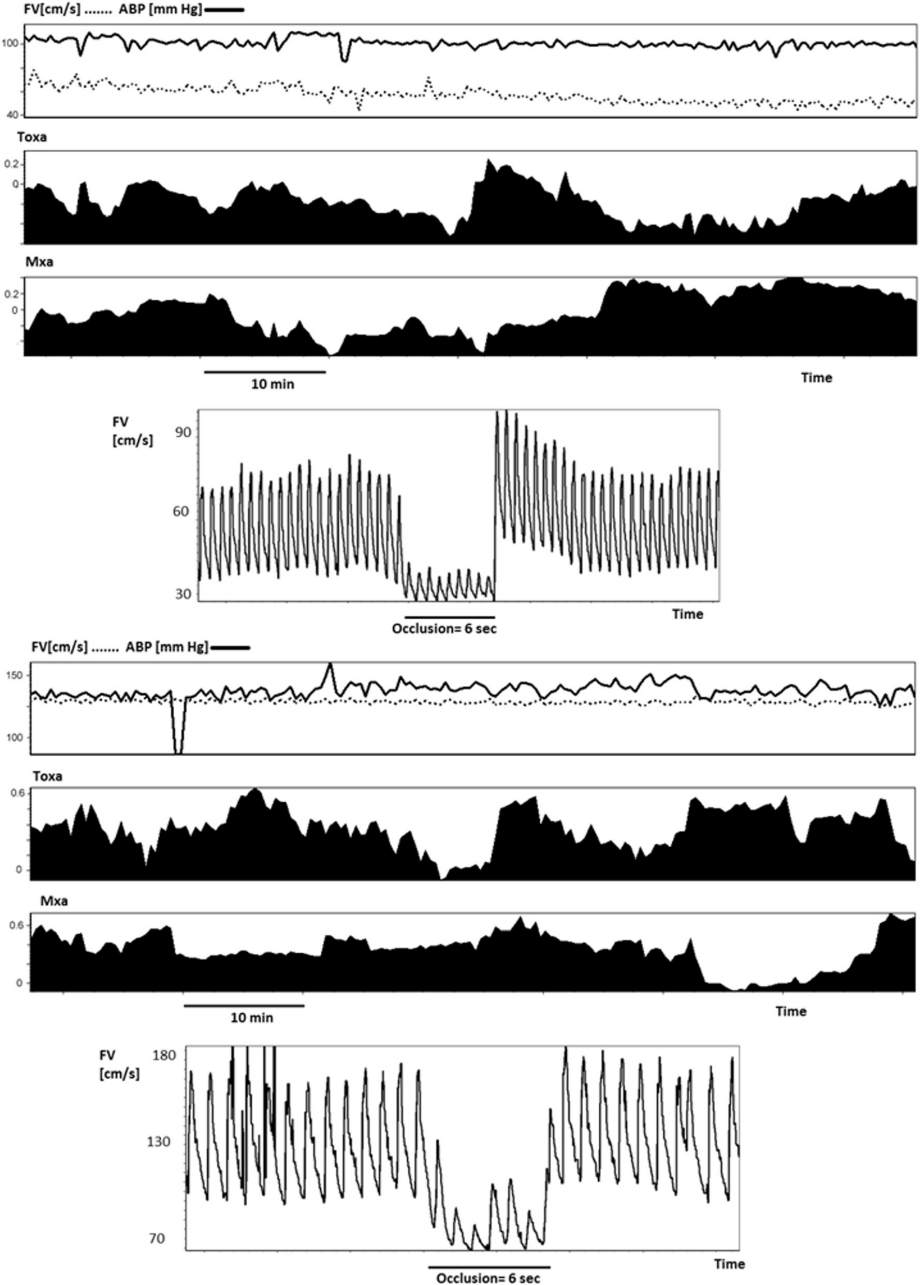
Cerebral perfusion monitoring in SAH. On day 3 after ictus (top 4 panels), this patient with SAH from an aneurysm of the middle cerebral artery displays a normal middle cerebral artery Fv (~60 cm/s) and intact autoregulation (TOxa and Mxa ~0 (suffix ‘a’ indicates that ABP is used instead of CPP)). On day 7 (bottom 4 panels) a marked increase in Fv (to 120 cm/s) can be seen, which is accompanied by an impairment in autoregulation (TOxa and Mxa close to 0). The transient hyperaemic response test also failed to show an increase in Fv after the release of occlusion, an indicator of impaired cerebral autoregulation. *ABP* arterial blood pressure, *Fv* flow velocity, *Mxa* mean flow index (with ABP), *TOxa* total oxygenation reactivity index (with ABP)

The transient hyperaemic response test is an alternative form of cerebral autoregulation testing which involves assessing the increase in TCD blood flow velocity after release of a short (5–10 s) compression of the common carotid artery [42]. The degree of increase in blood flow velocity in the seconds following release is thought to be a reflection of the extent of cerebral vasodilation in response to the reduced CPP during occlusion. An example of a transient hyperaemic response test is shown in Fig. 4.

In some cases, cerebral autoregulation can be estimated using ICP as a surrogate for cerebral blood volume. In this method, similarly to Mx, 30 consecutive 10-s averages of ABP are correlated with ICP to yield the pressure reactivity index (PRx) [40]. A positive correlation indicates passive transmission of ABP waves to cerebral blood volume and hence ICP, while a negative correlation indicates active counter-regulatory adjustments of the cerebrovasculature and intact vasoreactivity. PRx has the advantage that it can be easily measured continuously in any patient with a parenchymal ICP monitor, an arterial pressure line, and the appropriate analysis software.

From a critical care perspective, the assessment of cerebral autoregulation can be more practical than monitoring CO_2_ reactivity because we can utilise the natural fluctuations of ABP and therefore monitor cerebral autoregulation continuously. From a practical point of view, to monitor cerebral autoregulation requires a continuous estimate of CBF (NIRS or TCD are ideal), ABP (from an arterial line or photoplethysmography), and in some cases ICP.

Given the heterogeneity of CBF monitoring techniques and the versatility of signal processing techniques, a multitude of ‘indices’ or metrics of cerebral autoregulation have been proposed. Table 2 highlights the rationale of such indices and gives an opinion as to their usefulness.

**Table 2:** Summary of autoregulation indices

Autoregulation metric	Input signals	Calculation	Interpretation	Comment
Autoregulation index (ARI)	ABP, Fv	Compares the CBF response to changes in ABP with those predicted from a parametric model with 10 different ‘strengths’ of autoregulation [110]	ARI = 0 absent autoregulation, ARI = 9 perfect autoregulation	Moderately complex signal processing required
Flow index (Mx, Sx, Dx)	ABP (CPP), Fv	Pearson correlation between CPP and mean Fv (300-s window of 10-s averages). Sx and Dx calculated with systolic and diastolic flow velocity, respectively	Impaired autoregulation = higher Mx, Dx, and Sx	Simplistic yet prognostically relevant
Transfer function (phase, gain, coherence)	ABP, Fv	Derived from the transfer function of fast Fourier transform of ABP and Fv signals. Phase is the shift required to align Fv and ABP signals, gain the transmission from ABP to Fv, and coherence the statistical association between ABP and Fv	Impaired autoregulation = low phase, high gain, high coherence	Moderately complex signal processing. Some prognostic relevance
TOx, COx, THx, HVx	ABP (CPP), NIRS oxygenation	Pearson correlation between 30 consecutive 10-s means of ABP and tissue oxygenation (or total haemoglobin for THx and HVx)	Impaired autoregulation = higher TOx, COx, THx, HVx	Correlated with TCD methods but allows for longer term monitoring
TOIHRx	HR, NIRS oxygenation	Correlation between 30 consecutive 10-s means of HR and NIRS oxygenation	?Higher TOIHRx = impaired autoregulation	Used in preterm infants. Further comparisons with standard autoregulation indices required
Transfer function (phase, gain, coherence)	ABP, NIRS oxygenation	Derived from the transfer function of fast Fourier transform of ABP and oxygenation signals. Phase is the shift required to align oxygenation and ABP signals, gain the transmission from ABP to NIRS oxygenation, and coherence the statistical association between ABP and NIRS oxygenation	Impaired autoregulation = low phase, high gain, high coherence	Moderately complex signal processing
PRx	ABP, ICP	Correlation between 30 consecutive 10-s means of ABP and ICP	Higher PRx = impaired autoregulation	Robust measure for long monitoring periods. Simplistic and prognostically relevant
PAx	ABP, amplitude of ICP	Correlation between 30 consecutive 10-s means of ABP and ICP	Higher PAx = impaired autoregulation	Similar to PRx, may allow better estimate of pressure reactivity when the “pressure–volume” compensatory curve is flat, i.e. at low ICP
ORx	CPP (ABP), P_B_TO_2_	Correlation between 30 consecutive 10-s means of ABP and P_B_TO_2_	High ORx = impaired autoregulation	Further validation required

*ABP* arterial blood pressure, *ARI* autoregulatory index, *CBF* cerebral blood flow, *COx* cerebral oximetry index, *CPP* cerebral perfusion pressure, *Dx* diastolic flow index, *Fv* flow velocity, *HR* heart rate, *HVx* haemoglobin volume reactivity index, *ICP* intracranial pressure, *Mx* mean flow index, *ORx* oxygen reactivity index, *PAx* pressure amplitude index, *P*_*B*_ *TO*_*2*_ pressure of brain tissue oxygen, *PRx* pressure reactivity index, *Sx* systolic flow index, *NIRS* near-infrared spectroscopy, *TCD* transcranial Doppler, *THx* total haemoglobin reactivity index, *TOIHRx* total oxygenation heart rate index, *TOx* total oxygenation reactivity index

#### Neurovascular coupling

The increase in CBF accompanying cerebral cortical activation represents a further way of assessing the reactivity of vessels. Neurovascular coupling can be assessed with either TCD or NIRS to detect increases in CBF in response to cognitive, emotional, sensory, and motor tasks (for a recent review, see [18]). Although less studied than pressure or CO_2_ reactivity in the critical care population, neurovascular coupling assessment has great potential because it can be assessed non-invasively and repeatedly, and it reflects a physiologically distinct aspect of CBF regulation compared with CO_2_ or pressure reactivity.

### Clinical applications of bedside assessment of CBF regulation

Using the methodologies described, the cerebral circulation can be assessed in the critically ill patient. In this particular setting, techniques such as TCD, NIRS, ICP, and ABP monitoring are desirable as they can provide a continuous assessment of cerebral circulation without the need for transporting the patient. Unfortunately, validated ‘normal’ reference ranges are seldom available for the cerebral circulation and interpretation must therefore take into account relevant patient comorbidities and the underlying physiologic milieu. In the following section we summarise the role of the cerebral circulation in TBI, SAH, stroke, sepsis, and prematurity.

#### Traumatic brain injury

The pathophysiology of TBI is classically split into two phases, with the primary injury occurring at the time of ictus and secondary injury occurring in the following minutes, days, or even weeks. A cascade of pathophysiologic events leads to altered cerebral and systemic physiology that adds insult to injury; derangements in glucose metabolism, thermoregulation, respiration, and cerebral blood circulation all contribute to neuronal injury [43].

**Fig. 5:**
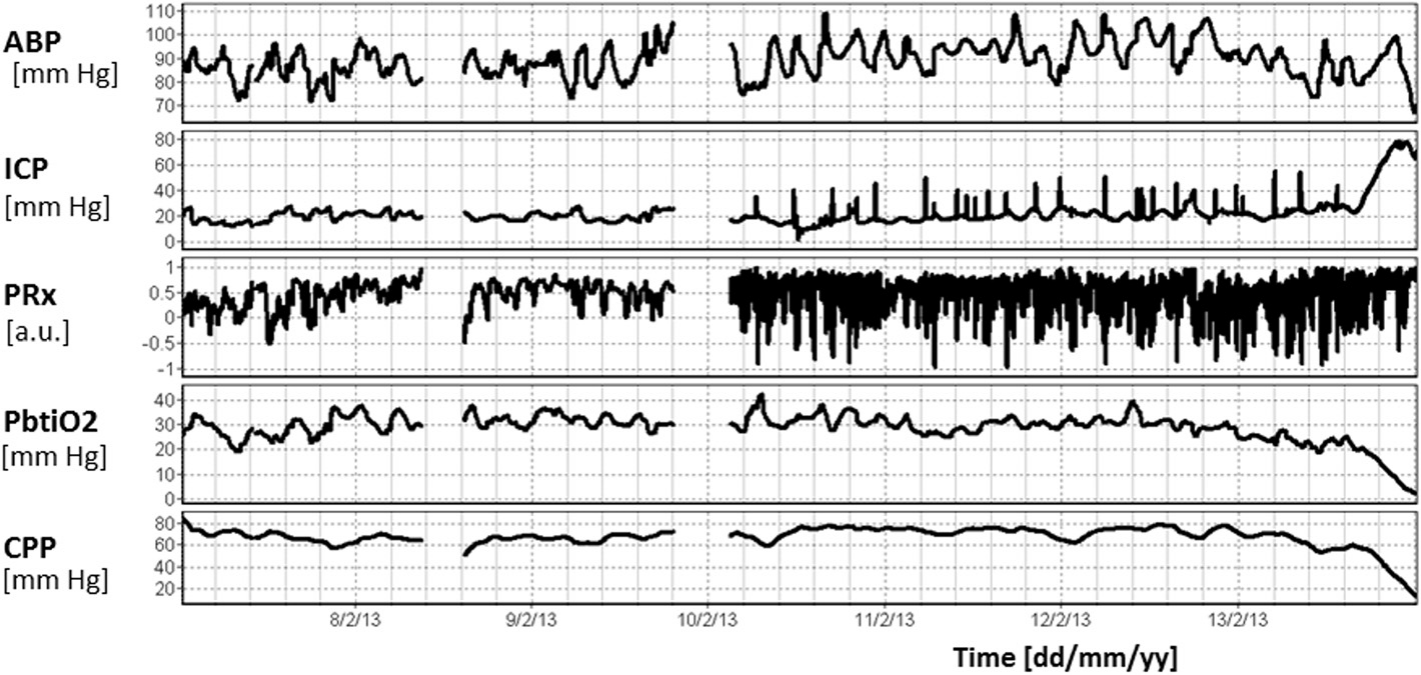
Continuous cerebral autoregulation monitoring during refractory intracranial hypertension. Continuous monitoring of cerebral autoregulation using PRx in a patient after severe TBI, who died after 6 days because of refractory intracranial hypertension. During the first 3 days ICP was stable, around 20 mmHg. However, PRx showed good autoregulation only during the first day (PRx <0.3). Later PRx was consistently above 0.5 even if ICP, CPP, and brain tissue oxygenation (*PbtiO*_*2*_) were satisfactory. After day 4, PRx was persistently elevated to >0.7. On day 6, ICP increased abruptly to 70 mmHg, CPP fell to 20 mmHg, and oxygen tension fell below 5 mmHg. The patient died in a scenario of brain-stem herniation. The only parameter which deteriorated early in this case was the index of cerebral autoregulation PRx. *ABP* arterial blood pressure, *CPP* cerebral perfusion pressure, *ICP* intracranial pressure, *PRx* pressure reactivity index

The characterisation of the cerebral circulation after severe TBI is not straightforward partly because the disease entity itself is heterogeneous. Despite this diversity, it is clear that maintaining close attention to cerebral perfusion is essential in all patients. The cerebral circulation is universally compromised after severe TBI; CBF, CO_2_ reactivity, and cerebral pressure autoregulation can all be impaired at various stages after TBI (Table 3). Low CBF, high CBF [44–46], and impaired autoregulation [47, 48] have all been associated with worse outcome (an example of temporal variations in CBF regulation in a TBI patient is shown in Fig. 5). However, while impaired CO_2_ reactivity has been shown to be related to unfavourable outcome in some studies [49, 50], this is not universal. Carmona Suazo et al. [51] used parenchymal brain tissue oxygen monitors to assess CBF in 90 TBI patients and found that, while all patients seemed to have a low CO_2_ reactivity on day 1, this gradually improved over the first 5 days of monitoring. Interestingly, CO_2_ reactivity on day 5 was higher in those with an unfavourable outcome. Unfortunately, a low sample size (*n* = 10 by day 5 of monitoring) and the potential for confounding changes in CPP make the generalisability of this surprising result uncertain.

**Table 3:** Cerebral haemodynamics in critical illness

Critical illness	Effect of disease on cerebral haemodynamics			Does the cerebral haemodynamic parameter relate to prognosis?		
	Flow	Cerebral autoregulation	CO_2_ reactivity	Flow	Cerebral autoregulation	CO_2_ reactivity
TBI	Decreased [45, 46, 111] then increased [46, 112]	Decreased [44, 113]	Decreased [44, 49, 114, 115]	Yes: decreased [44–46, 111] and increased [44, 112] CBF related to poor outcome	Yes [44, 47]	Most studies find yes [44, 49], while some find no [51]
SAH	Decreased (vasospasm) [14, 55]	Decreased [54, 56]	Decreased [55]	Yes [62]	Yes [60, 62, 116]	Yes [117]
Stroke	Decreased [66, 67, 118]	Decreased [70, 71]	Decreased [68, 69]	Yes [66, 67]	Yes [71]	Yes [69]
Sepsis	Unchanged [78, 81], or decreased [5]	Unchanged [82], decreased [78, 79], or increased [83, 84]	Unchanged [82] or decreased [77]	Unknown	Unknown	Unknown
Preterm infants	Decreased [87, 89, 119]	Unchanged [93] or decreased [87, 88]	Decreased [88, 90]	Yes [119]	Yes [88, 95, 120]	Yes [88, 90]

*CBF* cerebral blood flow, *CO*_*2*_ carbon dioxide, *SAH*, subarachnoid haemorrhage; *TBI*, traumatic brain injury

Given that CBF seems to show a distinct time evolution after TBI [46, 52], defining an optimal CBF is clearly problematic because it is likely to vary with the patients’ individual physiologic milieu, as well as the temporal evolution of disease. Furthermore, continuous measurements of CBF, although possible, are seldom feasible (Table 1) and therefore ICU therapies dictate not CBF per se but a target range of CPP. In this regard, individually optimising CPP to a continuously calculated measure of vascular reactivity such as PRx seems promising. The CPP dependence of PRx can be used to assess at which CPP the autoregulation is most efficient (i.e. the CPP at which the PRx is most negative). This is potentially important because CPP is a variable (unlike CBF or indices of autoregulation) that can be titrated precisely at the bedside. Importantly, the difference between CPP and the optimal CPP has been shown to be related to outcome [53].

**Fig. 6:**
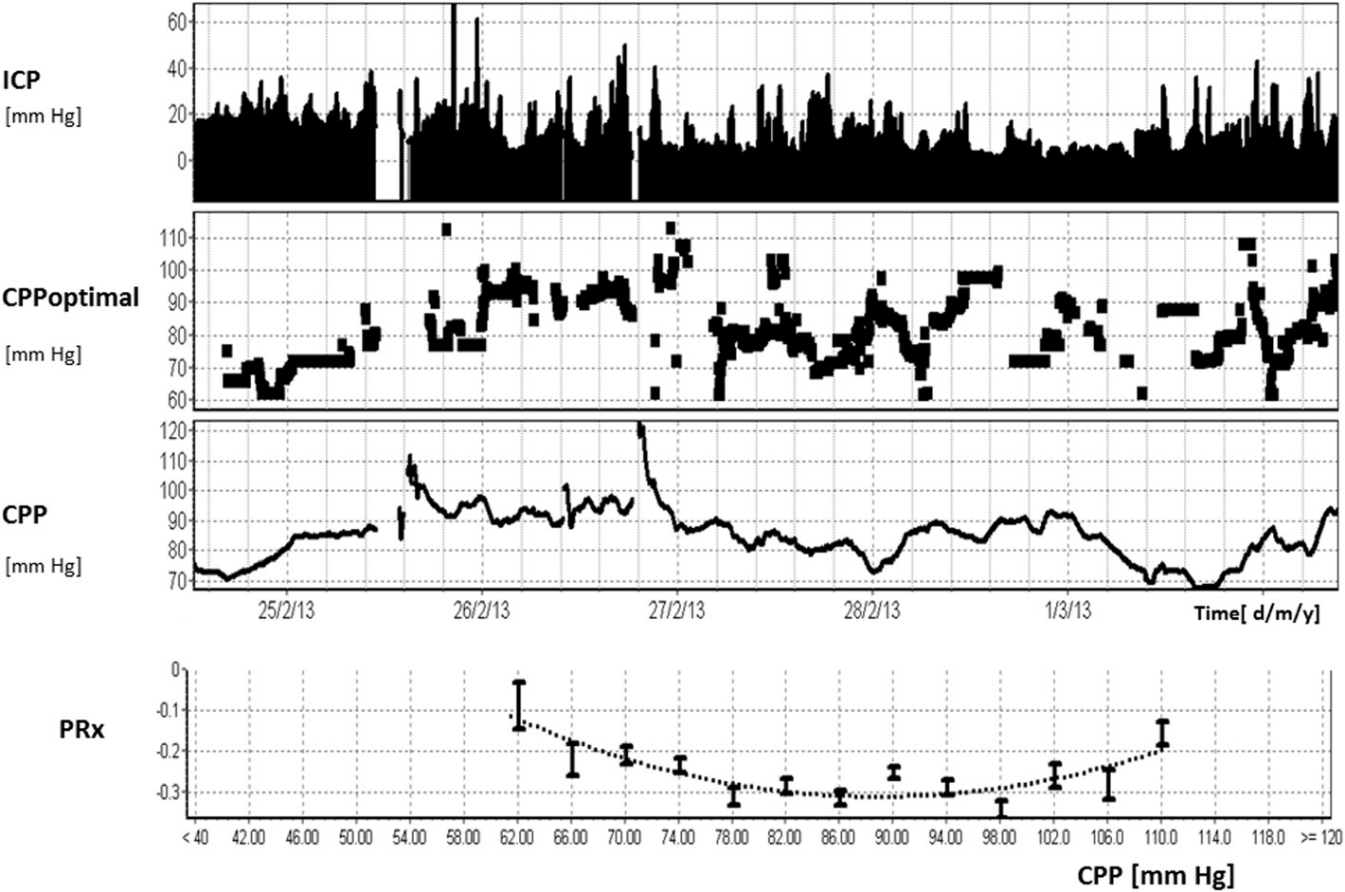
Long-term monitoring of PRx in a patient after TBI. ICP was first elevated to 20 mmHg and then decreased, showing some fluctuations over 7 days of monitoring. PRx had parabolic distribution along the recorded range of CPP (from 60 to 100 mmHg). The minimum of this parabola indicates ‘optimal CPP’ from the whole 7-day period (90 mmHg in this case—as compared with above 65–70 mmHg, advised by guidelines—which illustrates well that CPP-oriented management must be individualised; it is not true that one shoe size is good for everybody). Moreover, such a fitting of an ‘optimal curve’ may be repeated in time, based on data from the past 4 h. This enables prospective detection and tracing of ‘optimal CPP’ and targeting current CPP at its current optimal value, which may change in a course of intensive care. *CPP* cerebral perfusion pressure, *ICP* intracranial pressure, *PRx* pressure reactivity index

Figure 6 demonstrates long-term continuous monitoring of cerebral autoregulation using PRx in a TBI patient. In this case, ICP was initially above 20 mmHg and then subsided. The CPP varied between 60 and 100 mmHg, and when this CPP was plotted against PRx a U-shaped, parabolic curve is observed with a minimum at ~90 mmHg.

#### Subarachnoid haemorrhage

Spontaneous SAH most commonly results from rupture of an intracranial aneurysm. Following SAH, severe disturbances of CBF as well as CBF regulation can occur. These are frequently related to large vessel spasm, but may also be a sequelae of CBF dysregulation and a host of other pathological processes, such as cortical spreading depolarisations, acute inflammation, and loss of blood–brain barrier—all of which have been implicated in patient prognosis.

Early studies of experimental SAH in baboons revealed impaired CBF, CO_2_ reactivity, and cerebral autoregulation [54, 55]. However, like TBI, the clinical course of SAH is heterogeneous, especially with respect to CBF. Approximately 60 % of SAH cases develop vasospasm on TCD, which may be accompanied by impaired CBF and cerebral autoregulation [14, 56], and 15–30 % develop delayed ischaemic deficits [57–59]. While the relationship between vasospasm, delayed cerebral ischaemia, and outcome can be capricious, various aspects of cerebral haemodynamics can be useful in predicting the future clinical course: early impaired CO_2_ reactivity predicts vasospasm, and impaired cerebral autoregulation predicts delayed ischaemic deficits and poor clinical outcome [60, 61].

While CBF is typically within normal limits early after ictus, it is possible to see impaired cerebral autoregulation within the first 3–5 days after SAH [58, 60, 62]. Furthermore, Jaeger et al. [60] demonstrated that autoregulation can recover following the initial deterioration, a response that indicates a good prognosis. Figure 4 demonstrates the time course of CBF regulation changes in a patient after SAH.

Management strategies hinge on the early identification of delayed cerebral ischaemia, followed by the institution of hypertension to maintain CBF. Currently, nimodipine remains the only medication approved for prevention of delayed cerebral ischaemia. In this respect, optimisation of ABP according to cerebral autoregulation may be a promising avenue of research [63].

#### Ischaemic stroke

Ischaemic stroke is characterised by luminal obstruction by a blood clot. Thus, a region of the brain has abnormally high resistance and decreased flow (Fig. 1). In these patients, utmost importance is placed on prompt dissolution of the clot either by thrombolysis or intravascular clot removal [64]. Around the central core of infarct is a zone of tissue with depleted, but not absent, blood flow—the ischaemic penumbra. Prompt dissolution of the clot can salvage this at-risk tissue.

Unlike TBI, or SAH, a predisposition for ischaemic stroke can be determined by examination of cerebrovascular regulation; those patients with impaired CO_2_ reactivity are more likely to develop an ischaemic stroke [65]. However, like TBI and SAH, ischaemic stroke is a state where careful consideration of cerebrovascular regulation in the acute phase is imperative (Table 3).

In the acute phase of ischaemic stroke, those patients with the lowest global CBF tend to have worse prognosis [66], as do those with a greater proportion of penumbral to ischaemic tissue [67]. CO_2_ reactivity is depressed compared with healthy controls [68, 69] and those with lower CO_2_ reactivity have worse outcome [69]. Cerebral autoregulation also appears to be impaired initially, followed by further impairment over the ensuing several days before again improving (reviewed in [70]). In 45 ischaemic stroke patients, cerebral autoregulation impairment was related to both the size of infarct and functional outcome [71].

Ongoing controversy exists regarding how best to support the cerebral circulation after efforts to break down the intramural obstruction. While the prevention of hypotension after ischaemic stroke seems logical, knowledge of cerebral autoregulation has potential to help guide the management of blood pressure. Studies of continuous vascular reactivity are limited after ischaemic stroke because these patients are often managed outside the critical care environment without the insertion of invasive ABP or cerebral perfusion monitors that allow for continuous estimation of cerebral autoregulation. In this regard, non-invasive perfusion assessment with NIRS and ABP with finger photoplethysmography are promising.

Common to large ischaemic stroke, TBI, and SAH is the occurrence of spreading cortical depolarisations. These waves of near-complete depolarisation propagate slowly through the cortex (over a time scale of about 1 min) and are followed by several minutes of markedly depressed electrical activity [72, 73]. Their occurrence in an injured brain may decrease CBF, resulting in areas of ischaemia, and seem to lead to worse outcomes [74]. Whether they are a cause or a consequence (or both) of altered cerebrovascular regulation needs further investigation with simultaneous CBF circulation and electrocortical monitoring.

#### Sepsis

The host response to infection—sepsis—is characterised by dysfunction of multiple organ systems, including the brain. This host response can have implications for CBF: CPP is often low, pyrexia can alter CBF, and inflammatory mediators can alter vascular resistance [75, 76]. Compared with the aforementioned diseases, the cerebral circulation in sepsis is less completely characterised.

Some studies have found impaired CO_2_ reactivity [77], impaired autoregulation [78–80], and decreased CBF [5] during sepsis, whilst other studies have found no significant changes in CO_2_ reactivity, cerebral autoregulation, or CBF [81, 82]. Interestingly, two groups have even found that, in the early phases of experimental sepsis in healthy volunteers, dynamic cerebral autoregulation is actually enhanced [83, 84]. Pfister et al. [78] found that autoregulation was impaired in those with sepsis and delirium, but not in those with sepsis only. These seemingly conflicting findings may be partially explained by the heterogeneity of the sepsis process itself. Some septic patients develop a hyperdynamic circulation with increased cardiac output and decreased ABP, while others have both decreased cardiac output and ABP. Moreover, the physiological changes in the cerebral circulation during sepsis probably evolve over time, thus making comparisons between different studies difficult.

Nevertheless, brain dysfunction is one of the earliest forms of organ dysfunction in sepsis and sepsis-induced delirium occurs in up to 70 % of patients [76]. Characterising the involvement of the cerebral circulation in the pathogenesis of sepsis-induced delirium will probably require detailed haemodynamic studies with large numbers of patients.

#### Preterm infants

Premature infants do not have fully functioning cerebral vessels or cardiovascular systems and therefore vital organ perfusion is vulnerable. Using NIRS and umbilical artery ABP, continuous measures of cerebral autoregulation can be obtained.

Animal studies indicate that cerebral autoregulation starts to develop from around halfway through the gestational period [85]. Furthermore, even when static autoregulation is developed, the preterm newborn probably sits close to the lower limit of autoregulation [86]. Early human investigations using Xe CT and NIRS indicated that CBF, CO_2_ reactivity, and cerebral autoregulation may be impaired in preterm infants [87–91] (Table 3). Further, more recent human data using TCD indicated that cerebral autoregulation is more impaired if the baby is more premature [92]. Still other studies have indicated that perhaps the premature brain is able to adapt to sustained [93] but not dynamic [94] changes in ABP; that is, ‘static’ autoregulation is intact, while ‘dynamic’ autoregulation may be impaired [91].

Analogous to TBI, determination of an optimal ABP has been attempted in these preterm infants with the finding that those who did not survive had an ABP below their calculated optimal, whereas those who developed periventricular haemorrhage had an ABP above their optimal [95]. An important consideration when interpreting studies on cerebral haemodynamics in infants is that, in addition to the influences of ABP and CO_2_ on CBF, arterial oxygen saturation can be highly variable, and can have profound influence on premature babies’ cerebral circulation [96].

## Future directions

With the increasing availability of bedside physiology monitors and sophisticated online analysis software, large-scale integrated interrogations of CBF regulation are now possible. One important research theme is developing robust prediction tools based on cerebral physiologic monitoring for critically ill patients. Accurate prognosis is of obvious importance for patients, families, and clinicians alike, but current methodologies have some limitations. For example, prognostic tools in TBI use clinical, laboratory, and radiographic features on admission to predict patient outcome [97]. However, some of the input variables are open to interpretation (e.g. the grading of a CT scan), and prognosis should ideally be updated based on clinical and physiological developments. In this sense, prognostic tools that update risk estimates based on online monitoring of CBF regulation could facilitate clinical decision-making.

In addition to predicting outcome, incorporating knowledge of CBF regulation into management protocols seems promising. Hopeful examples in TBI include strategies that incorporate knowledge of cerebrovascular reactivity into either ICP [98] or CPP [53] management. Although still requiring further development and prospective assessment, similar techniques could conceivably be applied to any condition where ABP or CBF regulation is impaired.

**Fig. 7:**
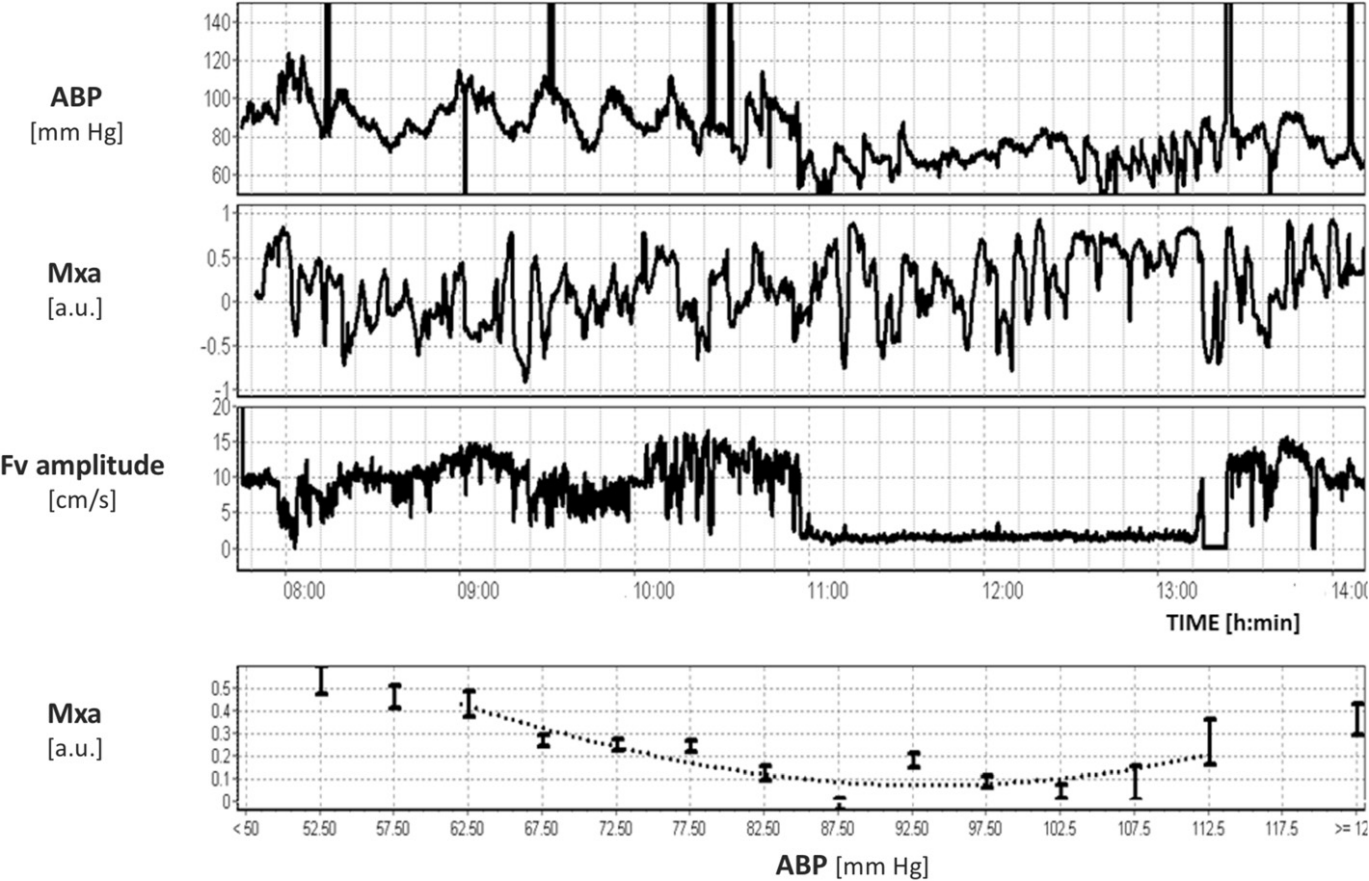
Monitoring of cerebral autoregulation during cardiopulmonary bypass surgery (re-analysis of raw data recording reported by Brady et al. [100]). TCD-derived autoregulation index Mxa fluctuates seemingly in a chaotic manner during surgery (period of laminar flow is denoted by near-zero pulse amplitude of the Fv waveform). However, its distribution along recorded blood pressure values resembles a parabolic curve—the same as seen in TBI patients—with its minimum indicating hypothetical ‘optimal’ blood pressure (in this case 96 mmHg). Adapted with permission of Prof. Charles Hogue and co-workers (John Hopkins Medical University) [100]. *ABP* arterial blood pressure, *Fv* flow velocity, *Mxa* mean flow index (with ABP)

Extending cerebral haemodynamic monitoring concepts to other critical care pathologies is important. For example, in cardiopulmonary bypass patients, NIRS-based autoregulation has been shown to be a significant predictor of outcome, and furthermore, as in TBI, an autoregulation-based optimal ABP seems to be prognostically important [99, 100]. An example of autoregulation-based optimal ABP during cardiopulmonary bypass is shown in Fig. 7. Cardiac arrest, acute shunt blockage, acute liver failure, pre-eclampsia, and malignant hypertension are all conditions that could perturb the cerebral circulation, and further investigation may reveal diagnostic, prognostic, or therapeutic insight.

Understanding the factors that modify CBF and vascular reactivity is also an important evolving area of research. Although a large part of the variation in cerebral autoregulation is accounted for by the level of ABP (or CPP) itself, other systemic and local factors may also be important. Preliminary investigations indicate that common occurrences in the critical care unit such as hyperglycaemia, altered renal clearance, erythrocyte transfusion, or rewarming after hypothermia are all associated with altered cerebral pressure reactivity, underscoring the need for an integrative approach to neuromonitoring [101–104].

Finally, investigating and integrating additional aspects of CBF regulation into prognostic and therapeutic approaches is imperative. In particular, the computerised assessment of neurovascular coupling [18] and autonomic function (e.g. with baroreceptor sensitivity or heart rate variability) are non-invasive, provide unique information on the regulation of CBF, and can be coupled with conventional measures of CBF regulation such as cerebral autoregulation and cerebrovascular CO_2_ reactivity.

## Conclusions

To date, there is no randomised trial showing that monitoring the cerebral circulation improves care of neurological patients. The link between autoregulation status and possible treatment is not firmly established, but great hope is linked to the idea of treating patients with an ‘optimal CPP’ (TBI or SAH) or ‘optimal ABP’ regime (cardiac surgery, preterm infants, or conceivably sepsis). However, these methodologies still await prospective clinical studies.

With such a research focus on characterising brain function in health, it is a sad fact that in most cases our ability to monitor brain function and the cerebral circulation in the critically ill patient is rudimentary. Recent Neurocritical Care Society guidelines attempt to correct this situation [105]. With the maxim ‘time is brain’, a renewed focus on high-fidelity cerebrovascular monitoring is required—irreversible cerebral ischaemia can occur in a matter of minutes.

Progress in the neurocritical care of vascular diseases will probably also depend on moving away from broad assumptions or ‘one size fits all’ physiological targets; each patient brings a different physiology which should be catered for. Using continuous markers of vascular function has the potential to optimise therapy to the individual patient’s need. With the sophistication of signal processing and bioinformatic tools increasing exponentially, the challenge lies in successful integration of cerebral circulation monitoring paradigms at the bedside.

### Note

This article is part of a series on *Neurocritical care*, edited by Fabio Taccone. Other articles in this series can be found at http://ccforum.com/series/NCRC.

## Abbreviations

ABP: arterial blood pressure
CBF: cerebral blood flow
CO_2_: carbon dioxide
CPP: cerebral perfusion pressure
CSF: cerebrospinal fluid
CT: computerised tomography
CVR: cerebrovascular resistance
Fv: flow velocity
ICP: intracranial pressure
Mx: mean flow index
NIRS: near-infrared spectroscopy
PaCO_2_: arterial pressure of carbon dioxide
PCO_2_: pressure of carbon dioxide
PRx: pressure reactivity index
SAH: subarachnoid haemorrhage
TBI: traumatic brain injury
TCD: transcranial Doppler
